# Role of DNase Activity in Human Sperm DNA Fragmentation

**DOI:** 10.3390/biom14030304

**Published:** 2024-03-04

**Authors:** Jaime Gosálvez, Carmen López Fernández, Stephen D. Johnston, Javier Bartolomé-Nebreda

**Affiliations:** 1 Genetics Unit, Department of Biology, Universidad Autónoma de Madrid, Cantoblanco, 28049 Madrid, Spain; jaime.gosalvez@gmail.com (J.G.); mariadelcarmen.lopez@uam.es (C.L.F.); javier.bartolome@halotechdna.com (J.B.-N.); 2 School of Environment, The University of Queensland, Gatton, QLD 4343, Australia; 3 School of Veterinary Science, The University of Queensland, Gatton, QLD 4343, Australia; 4 Halotech DNA, Faraday 7 Planta, Oficina 1.08, Cantoblanco, 28049 Madrid, Spain

**Keywords:** male infertility, sperm DNA fragmentation, DNase activity

## Abstract

In this clinical era of intracytoplasmic sperm injection (ICSI), where a single spermatozoon is chosen for fertilization, the diagnostic functionality of the classical parameters typically associated with fertilization, such as sperm concentration, sperm motility, acrosome integrity, and mitochondria, is perhaps becoming less critical. In contrast, the contribution of sperm DNA quality to our understanding of the impact of male fertility within the context of ICSI is gaining increasing interest and importance. Even with respect to natural conception, high levels of sperm DNA fragmentation (SDF) in the ejaculate can adversely affect reproductive outcomes. However, the precise origin of SDF pathology in sperm cells is often ambiguous and most likely to be multifactorial. Hence, the genetic makeup of an individual, unbalanced REDOX processes, enzymatic activity, environmental and lifestyle factors, and even damage during sperm handling in the laboratory all operate in a unique and often synergistic manner to produce or induce sperm DNA damage. Surprisingly, the contribution of active enzymes as potential agents of SDF has received much less attention and, therefore, is likely to be underrated. This review highlights the roles of different enzymes related to the degradation of sperm DNA as possible effectors of DNA molecules in spermatozoa.

## 1. Introduction

While sperm DNA fragmentation (SDF) can have a profound adverse effect on male factor fertility [1], the specific origin of SDF in an individual patient is ambiguous, primarily as there are multiple factors that trigger this process. Sakkas et al. [2] identified six main mechanisms involved in the production of DNA damage, ranging from testicular to post-testicular localized effectors. Testicular damage involves defective maturation, abortive apoptosis, and DNA strand breaks, all of which are mediated by enzymatic interventions. Post-testicular damage is also associated with oxidative stress. The synergistic participation of these two mechanisms may go a long way toward explaining the etiology of SDF. While these cellular mechanisms may serve as the primary tools to induce DNA damage, other exogenous factors such as pathological conditions, environmental stressors, and lifestyle factors are also known to either facilitate and/or exacerbate sperm DNA integrity [3]. In this modern era of assisted reproductive technologies (ART), we cannot also rule out the negative impact of iatrogenic damage as a direct consequence of post-ejaculation semen manipulation or storage. Although the causes of this additional damage are related to the capacity of injurious effectors to cause DNA damage, mainly through the induction of oxidative stress (e.g., cryopreservation, temperature fluctuation, centrifugation, exposure to synthetic media, etc.), the genetic background of each species and individual, associated with the structure of the genes controlling protamine production, may also play an important species-specific role in susceptibility to iatrogenic DNA [4,5]. Hence, an individual sperm cell’s vulnerability to SDF is likely to be a consequence of its genetic design, tolerance to unbalanced REDOX and enzymatic activity related to chromatin structure during spermatogenesis, all of which are coincidentally influenced by a combination of environmental and lifestyle factors or factors associated with sperm manipulation in the laboratory. In this review, we shall focus on the less well-known potential role and mechanism of enzymes to produce DNA nicking or DNA degradation leading to SDF.

## 2. DNases

DNases are hydrolytic enzymes that cleave phosphodiester bonds between sugars and the phosphate backbone of DNA. These enzymes may exhibit endogenous or exogenous localization. Endogenous nucleases have important biological roles, including DNA replication, chromatin condensation, recombination, and DNA repair [6,7,8]. Additionally, these enzymes protect cells from the intake of exogenous DNA and have been implicated in cellular DNA metabolism [9]. Depending on whether DNA cleavage catalyzed by these enzymes occurs within or at the 5′ or 3′ ends formed after breakage of the DNA molecule, DNases are classified as endonucleases or exonucleases. There are two main families of DNases: DNase I and DNase II. Both families of DNases are endonucleases but have different biological and biochemical characteristics. The DNase I family of nucleases cleaves DNA, generating 5′-P and 3′-OH ends. DNase II nucleases hydrolyze the phosphodiester backbone of DNA molecules via a single-strand cleavage mechanism, producing 5′-OH and 3′-P ends [10,11]. The DNase I family comprises four enzymes: DNase I, DNase X, DNase γ, and DNAS1L2. DNase I nucleases are fully active in the presence of both Ca^2+^ and Mg^2+^ at neutral pH [12]. While DNase I and DNAS1L2 are secretory proteins, DNase γ and DNase X are retained intracellularly because of their C-terminal domains [13]. Members of the DNase II family are present in almost all tissues. They are present in lysosomes and are, therefore, active at an acidic pH without the need for cations as co-factors for their activity [10]. However, divalent cations such as Zn^2+^ and Cu^2+^ and monovalent cations such as Na^+^ (at high concentrations) inhibit DNase II activity [10,14,15]. 

DNase activity is regulated by substrate specificity to prevent undesired degradation of chromosomal DNA. Depending on their function, DNases have different subcellular localization, and in turn, depending on their subcellular localization, the same DNase may have different functions. Nuclease activity is regulated by stringent substrate specificity, confined localization, or potent inhibitors, which avoid unwanted or uncontrolled degradation of cellular DNA [16,17,18,19]. Some of these proteins are secreted and act as exogenous nucleases. DNase activity in the extracellular space can act on free-circulating DNA (fc-DNA). It has been shown that fc-DNA is first generated by cleavage inside the cells by intracellular DNases, and subsequently, DNA fragmentation continues by extracellular DNases [20]. Finally, bacterial DNA that is present after infection may be reabsorbed by living cells, resulting in additional inappropriate DNA information [21].

Mature spermatozoa have the capacity to bind and internalize exogenous DNA, and this ability has been exploited for sperm-mediated transgenesis in mice. However, it has been observed that the internalization of exogenous DNA induces the activation of a Ca^2+^-dependent nuclease that degrades both exogenous and sperm chromosomal DNA [22]. This study revealed that permeabilization of hamster spermatozoa with Triton X-100 in the presence of MgCl_2_ induced the degradation of sperm chromatin into 50 kb fragments. This indicates the existence of endogenous nuclease activity that degrades sperm DNA at the loops that bind protamine toroids, the ultimate level of sperm chromatin organization [23]. This nuclease activity, localized in the sperm nucleus, is dependent on both Ca^2+^ and Mg^2+^ and, apart from hamsters, is also present in mouse and human spermatozoa [24]. In mice, this nuclease activity is mediated by endogenous topoisomerase IIB in cooperation with extracellular nuclease [25]. The existence of nuclease activity in epididymal fluid and seminal plasma has also been confirmed in mice, being more abundant in semen [26].

Although the evolutionary origin of DNases remains controversial, one hypothesis proposes that DNases have arisen in eukaryotic organisms, along with phagocytosis, to facilitate bacterial DNA degradation, such that determining the quantity, distribution, and activity of nucleases in vivo will be important for our understanding of their physiological functions and roles in various cellular signalling pathways.

## 3. Histone–Protamine Replacement: Endogenous Enzymes

The main biological function of the sperm cell is the transmission of an intact copy of the paternal genome, so the preservation of sperm DNA is crucial not only for fertilization of the oocyte but also for normal embryonic development [27]. As spermatozoa are the only cell type designed to leave the body and must move through the relatively hostile environment of the female genital tract, sperm chromatin must be sufficiently compacted to fit into a nucleus that is 10% the size of a somatic cell. This level of DNA compaction protects and shelters the paternal DNA from damage during its journey to the oocyte. Within the seminiferous tubule of the testes, the sperm cell undergoes a remarkable molecular remodeling process known as spermiogenesis. Associated with spermiogenesis, a large portion of the chromatin’s histone is replaced by protamines. This almost universal process, which occurs throughout the animal kingdom, has evolved in order to achieve protection of the genetic content delivered to the next generation.

Protamines are basic proteins that are enriched in arginine and cysteine. Cysteines form intra- and intermolecular disulfide bonds in placental mammalian species (although absent in most sub-eutherian vertebrates). This facilitates the sperm chromatin to reach a high degree of compaction and stability. The basic unit of protamine-bound DNA is termed the toroid. Toroids are connected by less-condensed (non-protamine-bound) chromatin, termed the toroid linker region, which in turn binds to the nuclear matrix [28]. The histone–protamine transition is a complex process in which histone variants and specific histone modifications play essential roles in modulating chromatin compaction and higher-order chromatin structures [29].

For vertebrate species, the transition from histones to protamines occurs in the late spermatid stage, with two protamine genes (protamine 1 and protamine 2) primarily participating in this process. These newly incorporated proteins interact with DNA via a central arginine-rich DNA-binding domain. Disruption of this gene activity, which may affect both protamines, leads to male infertility associated with defective sperm maturation [30]. Histone–protamine replacement is mainly mediated by the endogenous nuclease topoisomerase II. This enzyme gives rise to DNA breaks and subsequently reduces the torsional stress associated with these histone–DNA interactions. Histone disassembly and chromatin remodeling with newly synthesized protamines to achieve maximum chromatin packaging and the absence of DNA breaks are crucial to ensuring that spermatozoa obtain their full fertilization capacity to produce viable offspring [27,31]. If DNA breaks persist, they accumulate mainly due to the absence of DNA repair in these cells [32]. Persistent DNA breaks are maintained in the sperm as single- or double-strand breaks in the DNA molecule, which produce defective chromatin packaging, resulting in flawed maturation. Ultimately, an increase in the level of sperm DNA fragmentation (SDF), with an inherent loss of sperm capacity for fertilization, is observed in neat ejaculate [33].

In parallel, abortive apoptosis during spermatogenesis typically prevents the defective chromatin from resulting in “quasi-normal” spermatozoa that might compete with normal spermatozoa for fertilization. If apoptosis is incomplete or defective, there will be an accumulation of spermatozoa expressing apoptotic markers in the ejaculate [34]. These processes occur in the presence of Fas ligands that bind to their receptors, which regulate apoptosis and other cellular processes of cell death in multiple cell types. One of the most important mechanisms that operates during sperm maturation is the inhibition of cell differentiation through a caspase-8-mediated mechanism. It has been proposed that assessment of Fas expression in ejaculated spermatozoa can be used as an indicator of elevated abortive apoptosis [35].

## 4. ROS Triggering Apoptotic Pathways

Given that different molecules, such as superoxide ion radicals, hydrogen peroxide, hydroxyl radicals, alkoxyl radicals, nitric oxide, or their derivatives, may act as stressors for DNA, proteins, and lipids, an imbalance between the production of free radicals and antioxidant capacity can produce severe injuries to the sperm cell at different levels (DNA, proteins, and lipids). When this occurs, apoptotic pathways are activated in the spermatozoa [36]. Moreover, SDF can be directly produced by products such as malondialdehyde and 4-hydroxynonenal, which are related to lipid peroxidation. These reactions may produce DNA adducts, such as 8-hydroxy-2′-deoxyguanosine (8-OHdG), 1, N6-ethenoadenosine, and 1, N6-ethenoguanosine, resulting in a non-orthodox state of the DNA and thereby compromizing normal genetic function [37,38,39]. The incorporation of adducts such as 8-OHdG is mainly concentrated in chromatin domains with relatively low-enrichment protamine regions [40].

Excessive pro-oxidant capacity may also activate the apoptotic pathways in spermatozoa. Apoptosis causes the externalization of phosphatidylserine localized in the sperm membrane; this structural and early marker of cell death can be used as a late marker of sperm DNA enzymatic degradation [36]. This process is completed by the proteolytic activation of caspases 3, 6, and 7, which are triggered via a mitochondria-mediated pathway with the participation of cytochrome C released into the cell [41,42]. Finally, and related to an excess of pro-oxidant capacity, the mitogen-activated protein kinases (MAPKs), which regulate different cellular programs by relaying extracellular signals to intracellular responses, may be triggered. Activation of the MAPK pathway increases p53 and caspase-3 expression and, in parallel, reduces the activity of regulating cell death genes, such as B-cell lymphoma 2 (BCL2) genes [43]. Synergic mechanisms among these factors may lead to impaired sperm maturation and promote apoptosis [44].

## 5. Presence of DNase Activity in the Ejaculate

**Figure 1:**
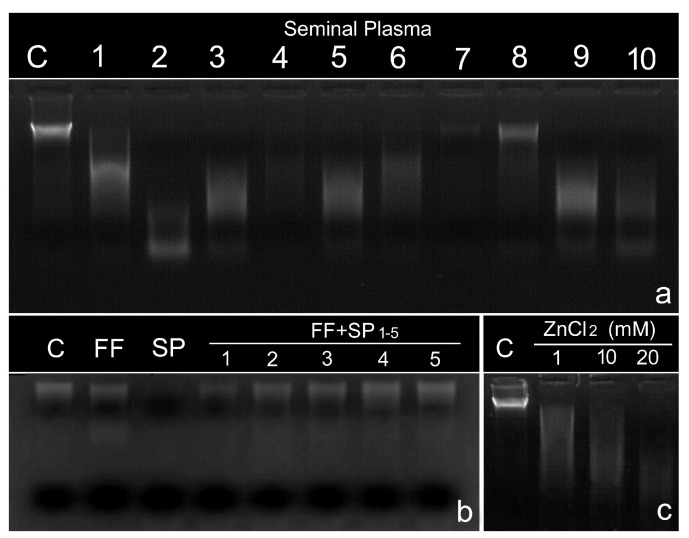
(**a**) DNase activity in seminal plasma. It can be observed that when genomic DNA is incubated with seminal plasmas, the intensity of genomic DNA degradation varies between the different plasmas. C, genomic DNA incubated with dH_2_O. (**b**) Inhibition of DNase activity in the seminal plasma (SP) by the follicular fluid (FF). As seen in the figure, the addition of FF to SP inhibits the degradation of genomic DNA. As controls, genomic DNA was incubated with dH_2_O (C), with follicular fluid alone (FF), and with seminal plasma (SP). (**c**) Effect of supplementation of seminal plasma with Zn^2+^. Seminal plasma with low basal DNase activity (C) was incubated with increasing concentrations of ZnCl_2_. As can be seen, the addition of Zn^2+^ induces the DNase activity of seminal plasma in a dose-dependent manner.

Dnase activity is also present in seminal plasma [45,46], although the intensity of this activity varies between the different seminal plasmas (Figure 1a and [47] in this paper). These enzymes can cross cell membranes and affect nuclear DNA. In a recent investigation, our group demonstrated that leukocyte DNA compacted with histone could be cleaved by Dnase activity, with the resulting DNA damage corresponding mainly to single-strand DNA breaks [48]. The transmembrane capacity of this enzyme may also be operative in the sperm DNA of the neat ejaculate and thereby increase the baseline levels of SDF in the ejaculate. This effect is reinforced when the entire neat ejaculate is stored in the laboratory for a prolonged period prior to artificial insemination or sperm cell injection [4]. Mammalian species typically ejaculate in different fractions, but in human ART, these fractions are typically mixed in a single vessel for collection; such mixing does not normally occur during natural insemination. The first fraction of the human ejaculate arriving at the cervix is typically enriched with spermatozoa compared to the second. Dnase activity has been shown to be significantly lower in the second fraction of the ejaculate when compared to the first [49]. While the biological and functional significance of this phenomenon is unknown, further research effort is required to better understand its physiological significance so that semen manipulation procedures in the laboratory can better reflect what occurs in nature, leading to improved assisted reproductive outcomes.

**Figure 2:**
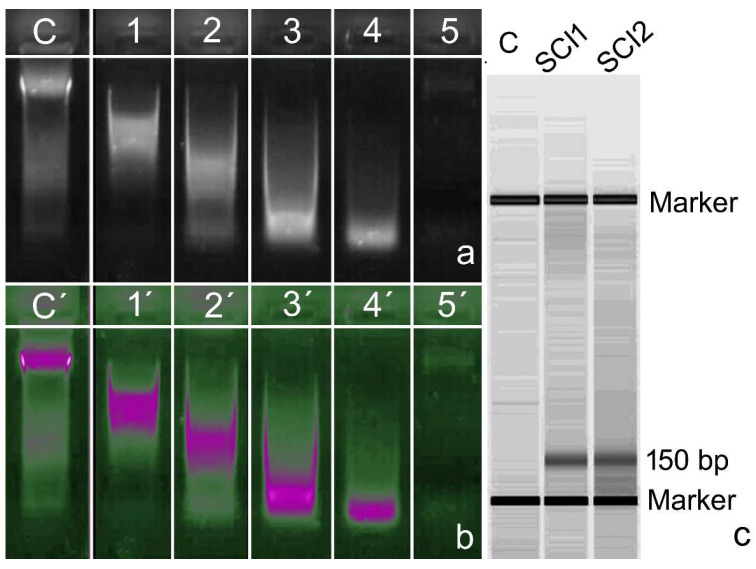
DNase activity in the human sperm-free seminal plasma. (**a**) From 1 to 5, a series of selected patients showed increasing intensity of DNA cleavage, as visualized by agarose gel electrophoresis using whole, isolated DNA. C: Control DNA was treated with distilled water. (**b**) An electronically filtered image showing a high concentration of DNA fragments produced after DNA cleavage (more details can be found in [41]). (**c**) Bioanalyzer results of cfDNA in patients with spinal cord injury (SCI1 and SCI2) compared with the control (C). Note the presence of a 150 bp DNA fragment in SCI patients (further details in [52]).

When DNase activity is tested in seminal plasma, its effectiveness in digesting naked DNA varies among individual seminal plasma samples ([45,46,48,49]; [Figure 2a,b]). Although it is clear that seminal plasma DNases are efficient at digesting naked DNA, the question that arises is whether these nucleases are capable of acting on DNA compacted in chromatin in the cell nucleus. In relation to this, our group has demonstrated, using leukocytes as target cells, that following incubation with seminal plasma induces the appearance of single-strand breaks in nuclear DNA, while incubation with recombinant DNase I causes the appearance of both double and single-strand breaks [48]. The effect of DNases from seminal plasma on chromatin as compact as that of sperm is currently under investigation by our group.

On the other hand, we recently reported the massive presence of DNase activity in patients with spinal cord injury (SCI), while the ejaculates of these patients also possess extremely high levels of sperm with DNA damage. While this was an interesting observation, it is difficult to attribute a causal relationship to this correlation because of other contributing and potentially confounding factors that might cause SDF in these individuals [50,51]. Interestingly, we also described the presence of cell-free DNA (cf-DNA), represented by two main DNA electrophoresis bands of 90 and 150 bp, in the majority of these same patients ([51]; [Figure 2b]). Consequently, within the same patient, we have high levels of SDF, high DNase activity in the seminal plasma, and a relatively large amount of cfDNA. If the presence of DNase activity was associated with high levels of SDF, then one might also assume the ability of these enzymes to remove this cf-DNA, but this is not the case.

Another aspect that has been poorly investigated in reproductive tissues is the presence of cfDNAs. cf-DNA is the product of dying cells or cell products related to the immune response and can be identified by the immune system, triggering or increasing the inflammatory response [53]. This reaction produces additional collateral effects because the inflammatory response is produced, in this case, via reactive nitrogen species. These biomolecules are potent pro-oxidants that interfere with normal and viable cells and spermatozoa [54]. Subsequently, effective cleavage of cf-DNA, which prevents or abolishes the inflammatory response, is key to maintaining homeostasis. Within this context, the presence of a certain level of DNase activity in the seminal plasma would be necessary since it would be required for the complete elimination of DNA motifs emerging from spermatozoa presenting a fragmented DNA molecule, and the iterative presence of SDF is an inherent feature of every ejaculate so far analyzed for this condition [4].

Additionally, cf-DNA with highly cohesive 5´-3´ends may act as potential DNA for sperm transfection. In animal models, such as the chicken, transgene transmission by sperm-mediated gene transfer after seminal plasma removal and exogenous, properly prepared DNA with dimethylsulfoxide or N,N-dimethylacetamide has been possible [55]. Spermatozoa with their own chromatin structures are more likely to include exogenous DNA pieces, given that they contain DNA domains with a high number of alkali-labile sites [56,57]. These DNA sites behave as abasic sites because they are susceptible to digestion by exonuclease III [58]. Spermatozoa, as valuable vehicles for gene transfer, were used for the first time by Brackett et al. [59] and are still considered potential cells for transgenesis [60].

**Figure 3:**
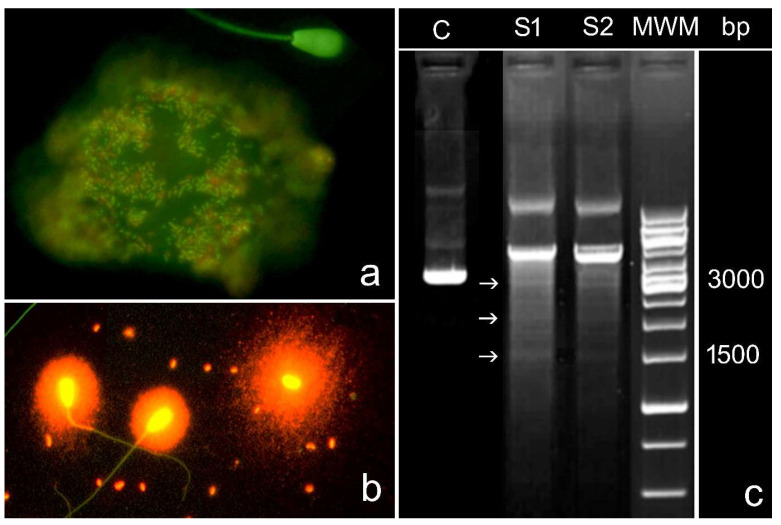
Presence and effects of bacteria on ejaculate. (**a**) Bull sperm ejaculate of an infertile male with a massive presence of endosymbiotic bacteria affecting the somatic cells present in the ejaculate. Cell membrane viability staining revealed the presence of dead (red) and live (green) bacteria. (**b**) Free sperm bacteria in cryopreserved bull samples. Sperm DNA fragmentation was assessed using a sperm chromatin dispersion assay (normal sperm: two cells on the left), sperm with fragmented DNA, and cells on the right that presented a large halo of dispersed chromatin. (**c**) Two infected cryopreserved samples of bull sperm (S1 and S2) after agarose gel DNA electrophoresis show the classical ladder of DNA fragments (arrows) produced after repetitive cleavage of DNA by restriction endonucleases produced by bacteria. (C) Control: bull DNA from one straw without bacterial presence. MWM: molecular weight DNA marker.

Other inducers of sperm DNA damage include bacterial infections of the male genital tract. They may have an internal origin, such as urogenital infections produced by *Chlamydia* or *Mycoplasma*, which may be present as endosymbionts and localized in somatic cells or free elementary bodies in the ejaculate (Figure 3a), or they may have an external origin, for example, bacteria inadvertently transmitted following artificial insemination of contaminated semen (Figure 2b). It has been shown that infection status is correlated with semen quality parameters, including DNA fragmentation [61,62,63,64], and that antibiotic treatment results in a decrease in the sperm fragmentation index [61,65]. The effect of bacterial infection on DNA fragmentation may be a consequence of an inflammatory reaction that induces oxidative stress owing to the release of ROS by leukocytes and the consequent induction of apoptosis, as explained above. Another mechanism by which bacterial infections may cause sperm DNA fragmentation is the induction of sperm apoptosis by lipopolysaccharides in the bacterial wall, as suggested by in vitro studies [64].

However, bacterial infections may lead to sperm DNA fragmentation via a more direct mechanism that uses self-produced restriction endonucleases. Our group has demonstrated that commercial doses of bull semen can be contaminated by bacteria, which increases the level of SDF via bacterial growth after cryopreservation and thawing [65]. This was evident when semen samples from the same bull with and without bacterial contamination were incubated at 37 °C, resulting in an increase in the rate of sperm DNA fragmentation in infected doses compared to uninfected doses [65]. Since bull semen samples were diluted in cryoprotective media, the likelihood that DNA fragmentation was due to the production of ROS by residual leukocytes in the samples is very low. Consequently, this increase in sperm DNA damage appears to be associated with the direct effect of contaminating bacteria. One possibility is that the increase in DNA fragmentation was due to the induction of apoptosis by lipopolysaccharides in the bacterial wall. Another possibility is that the fragmentation was due to the action of nucleases released into the medium by contaminating bacteria, as it is well known that bacteria are capable of releasing nucleases, including restriction endonucleases, into the medium [66,67]. In this case, a typical DNA ladder was present after massive DNA cleavage, and repetitive accumulation of DNA fragments was observed (Figure 3c). Co-incubation of the plasmid pBR322 with supernatants of commercial doses of bacteria-contaminated bull semen showed that the plasmid DNA was cleaved, and discrete DNA bands were observed after electrophoresis. This finding reinforces the idea that restriction endonucleases are present in the culture medium.

## 6. The Role of Follicular Fluid in Controlling DNase Activity

Seminal plasma nuclease activity appears to have been conserved from fish to mammals [68,69,70,71], suggesting that it plays an important physiological role in preventing the uptake of exogenous DNA by sperm [69]. Another function of seminal plasma nucleases may be related to the fact that an inflammatory-like response occurs in the female reproductive tract [72,73]. Neutrophils in the female genital tract are responsible for eliminating excess sperm and microbial contaminants by releasing to the extracellular space the so-called Neutrophil Extracellular Traps (NETs) formed by chromatin and proteins that engulf contaminating microorganisms [74,75]. These NETs can also trap sperm cells, reduce their motility, and consequently reduce their fertilizing capacity [76]. The physiological role of seminal plasma nucleases is to digest the DNA components of NETs, facilitating sperm motility and access to the ovum. In addition to these beneficial physiological functions, seminal plasma nucleases can be detrimental to fertilization because they can cause single-strand breaks in cells when chromatin protamination occurs [47,77]. Under this scenario, seminal plasma nucleases arriving in the oviduct may potentially cause DNA damage in fertilized oocytes. The higher than normal presence of nucleases in the female reproductive tract may be facilitated by ART practices such as artificial insemination; unanswered questions remain as to how harmful this might be and how it might be avoided in the future.

Follicular fluid (FF) is the microenvironment surrounding the oocyte within developing follicles. At ovulation, FF and the cumulus–oocyte complex are drawn into the fallopian tube so that FF is part of the normal milieu in which fertilization occurs. In the management of spermatozoa for assisted reproductive procedures, it has been shown that the supplementation of semen extenders with FF can result in better preservation of sperm chromatin structure and prolonged DNA integrity [46,47]. This beneficial effect of FF on sperm DNA may be related to the fact that FF inhibits the nuclease activity of seminal plasma (Figure 1b) [46]. This inhibitory effect is due to the fact that FF contains a molecule(s) that is capable of chelating the divalent cations necessary for the activity of nucleases not only in seminal plasma but also in the nucleases that are also present in FF itself or those directly inoculated during insemination [52]. While our experimental data suggest that seminal plasma nucleases play an important role in reproduction by protecting sperm from exogenous DNA and by facilitating sperm travel through the female reproductive tract, they could also jeopardize the integrity of fertilized eggs. We might speculate that evolutionary pressure has created an environment in which fertilization can inhibit these nucleases at the time of fertilization.

## 7. Antioxidant Therapy and Homeostatic Modifications

We suggest that uncontrolled therapeutic prescription of biomolecules to reduce oxidative stress can lead to adverse collateral effects on reproductive outcomes. For example, there has been growing interest in the potential use of L-carnitine as a nutritional/dietary supplement. This amino acid is thought to act as a direct scavenger of free radicals and chelates catalytic metal promoters such as Fe^2+^ and Cu^2+^, which are related to reactive oxygen species activity and, in turn, can inhibit oxidases such as NAPDH. In addition, L-carnitine helps to maintain mitochondrial integrity and prevents the formation of reactive oxygen species under stressful conditions. It also participates in the activation of antioxidant enzymes such as CAT, SOD, GSH-Px, GR, and GST [78]. Despite these powerful antioxidant properties, this amino acid has been shown to be contraindicated in patients diagnosed with varicocele, as it also causes dilatation of the arteries [79]. Hence, the administration of this compound with the idea of producing putative benefits is not recommended for all patients [80].

We have already noted that intra- or intercellular pro-oxidant capacity may damage DNA molecules. To abolish the REDOX imbalance, a series of antioxidant cocktails is often prescribed to patients when the level of SDF is high. These cocktails contain a series of vitamins, oligoelements, amino acids, and other compounds that interfere with the excess pro-oxidant capacity but may also inadvertently alter other pathways of metabolic utilization of resources, producing indirect and undesirable collateral effects. This was shown to be the case for Zn^2+^ supplements, a common oligoelement found in such prescribed antioxidant cocktails. A high concentration of Zn^2+^ may trigger DNase activity, and this alteration may subsequently translate to the DNA molecule [81]. Using animal models in which males were fed a diet with normal Zn^2+^ or only 200 parts per million of Zn-Methionate (50 ppm, over the recommended concentration), a significant increase in SDF was observed over the weeks following Zn^2+^ administration [82]; this effect disappeared after normal dietary recovery. While DNase activity in the seminal plasma of these individuals was not examined, a plausible hypothesis may be that the elevated level of SDF may have been associated with an increase in DNase activity since in vitro supplementation of seminal plasma with Zn^2+^ increases its DNase activity (Gosálvez J, unpublished data) (Figure 1c). The synergistic or adverse effects of prescribed biomolecules acting on such a complex process as “reproduction” are difficult to predict or manage.

## 8. Conclusions

Sperm DNA fragmentation must be considered a result of synergistic multifactorial processes leading to the production of non-orthodox DNA molecules in spermatozoa and impaired reproductive outcomes. Thus, the genetic design of the individual, REDOX-unbalanced processes, enzymatic activity affecting DNA proteins and lipids, environmental or lifestyle factors, and iatrogenic damage during sperm handling in the laboratory are all potential stress effectors that can produce sperm DNA modifications. In general, oxidative stress has been claimed to be one of the most influential effectors of sperm DNA damage, and while we agree, other biological biomolecules, such as enzymes, having both an endogenous and exogenous origin, may also modify the deoxyribonucleic acid molecule, producing irreparable DNA modifications in the sperm cell. The quantity and diversity of these enzymes associated with the reproductive system must not be overlooked in this modern era of assisted reproduction since they may also be significantly contributing to male infertility.

## Data Availability

Not applicable.
